# Recent Use of Novel Data Streams During Foodborne Illness Cluster Investigations by the United States Food and Drug Administration: Qualitative Review

**DOI:** 10.2196/58797

**Published:** 2025-02-28

**Authors:** Michael C Bazaco, Christina K Carstens, Tiffany Greenlee, Tyann Blessington, Evelyn Pereira, Sharon Seelman, Stranjae Ivory, Temesgen Jemaneh, Margaret Kirchner, Alvin Crosby, Stelios Viazis, Sheila van Twuyver, Michael Gwathmey, Tanya Malais, Oliver Ou, Stephanie Kenez, Nichole Nolan, Andrew Karasick, Cecile Punzalan, Colin Schwensohn, Laura Gieraltowski, Cary Chen Parker, Erin Jenkins, Stic Harris

**Affiliations:** 1 US Food and Drug Administration, College Park, MD, United States; 2 US Food and Drug Administration, Minneapolis, MN, United States; 3 US Food and Drug Administration, Silver Spring, MD, United States; 4 US Centers for Disease Control and Prevention, Atlanta, GA, United States

**Keywords:** foodborne illness surveillance, novel data streams, outbreak investigations, novel data, foodborne illness, foodborne, illness, United States, public health, prevention, outbreaks, social media, product review, cluster, product information, surveillance, epidemiology

## Abstract

Foodborne illness is a continuous public health risk. The recognition of signals indicating a cluster of foodborne illness is key to the detection, mitigation, and prevention of foodborne adverse event incidents and outbreaks. With increased internet availability and access, novel data streams (NDSs) for foodborne illness reports initiated by users outside of the traditional public health framework have emerged. These include, but are not limited to, social media websites, web-based product reviews posted to retailer websites, and private companies that host public-generated notices of foodborne illnesses. Information gathered by these platforms can help identify early signals of foodborne illness clusters or help inform ongoing public health investigations. Here we present an overview of NDSs and 3 investigations of foodborne illness incidents by the US Food and Drug Administration that included the use of NDSs at various stages. Each example demonstrates how these data were collected, integrated into traditional data sources, and used to inform the investigation. NDSs present a unique opportunity for public health agencies to identify clusters that may not have been identified otherwise, due to new or unique etiologies, as shown in the 3 examples. Clusters may also be identified earlier than they would have been through traditional sources. NDSs can further provide investigators supplemental information that may help confirm or rule out a source of illness. However, data collected from NDSs are often incomplete and lack critical details for investigators, such as product information (eg, lot numbers), clinical or medical details (eg, laboratory results of affected individuals), and contact information for report follow-up. In the future, public health agencies may wish to standardize an approach to maximize the potential of NDSs to catalyze and supplement adverse event investigations. Additionally, the collection of essential data elements by NDS platforms and data-sharing processes with public health agencies may aid in the investigation of foodborne illness clusters and inform subsequent public health and regulatory actions.

## Introduction

An estimated 1 in 6 people experiences an episode of foodborne illness in the United States annually, placing an estimated US $77.7 billion burden on the US economy [1,2]. Most of these illnesses are considered sporadic; however, limitations in foodborne outbreak surveillance may hinder the detection of outbreak-associated cases. The identification and investigation of foodborne illness clusters requires a high degree of coordination across state and local jurisdictions and, in some cases, federal agencies [3].

At the federal level, outbreaks are investigated when they involve cases in multiple states, implicate imported products, or when federal support is requested. Most of these outbreaks that involve known pathogens are traditionally initiated through laboratory-based surveillance systems with the identification of illness clusters by molecular subtyping of clinical isolates or case reports from ill individuals with a common exposure and illness who seek medical care. These clusters can then be linked to a common vehicle based on supporting epidemiologic information, traceback findings, and laboratory testing results from food and environmental samples. This information is usually first received by state or local health departments (LHDs) before it is passed on to federal partners, when deemed necessary. Outbreaks that involve known pathogens may also be identified through matching the genetic similarity of historic and contemporary isolates from product or environmental samples to clinical isolates via molecular subtyping techniques, such as whole genome sequencing [4].

In situations where an etiology is unknown or has not yet been identified, consumer complaint systems provide a mechanism for consumers to self-report adverse health events, including foodborne illness. This allows public health entities to conduct disease surveillance to help mitigate this primary limitation of laboratory-based surveillance. Consumer complaint–based systems allow for the collection and storage of consumer reports that can be submitted in a range of formats, including in-person, phone, e-mail, and web form [5]. A survey of LHDs in the United States revealed that a majority (81%) use consumer complaint–based systems and attributed the detection of 69% of foodborne outbreaks in the past year to consumer complaints [6]. State health departments that have conducted self-assessments of their consumer-complaint systems, such as Minnesota and Rhode Island, have credited the detection of 79% and 80% of foodborne outbreaks to consumer complaints, respectively [7,8]. Of the surveyed LHDs in the United States, 75% reported the ability to receive consumer complaints via email, and 40% reported the availability of a web-based reporting form [6]. The Integrated Food Safety Centers for Excellence help states to continually improve these complaint-based surveillance systems as well [9]. In the United States, the various consumer complaint systems create a patchwork that helps to capture information on foodborne illnesses that may not be reported to medical professionals. However, holistically, this patchwork can pose challenges as data from the various consumer complaint systems are stored separately. Recognition of signals depends on a sufficient number of complaints to come to the same complaint system.

Complaint-based surveillance is also used at the federal level to identify potential clusters of foodborne illness. The US Food and Drug Administration (FDA) collects complaints or reports submitted from consumers, health care providers, industry members, public health officials, or other submitters via 2 web-based mechanisms using the MedWatch or Safety Reporting Portal as well as by phone, email, fax, and physical mail [10-13]. A complainant may submit similar reports via multiple mechanisms, and it may not be possible to distinguish if they are duplicate reports or are separate cases. A key element of identifying illness clusters via this approach is the availability of complete and reliable epidemiologic data, such as illness onset date and contact information, to link complainant reports. This information is also needed to narrow down potential etiology and food vehicle [5].

Disease surveillance and outbreak information received by the FDA from various internal and external sources, including federal, state, and local health partners, is evaluated by the FDA’s Coordinated Outbreak Response & Evaluation (CORE) Network, which was established in 2011 and charged with coordinating efforts to find, stop, and prevent illnesses linked to FDA Center for Food Safety and Applied Nutrition (CFSAN) regulated products [14]. These products include all human foods not falling under the jurisdiction of the US Department of Agriculture Food Safety Inspection Service and account for 75% of the food supply in the United States [15]. Once an actionable signal has been identified, further investigational activities and public health actions (eg, product traceback, recalls, and communication) are coordinated across local, state, and federal partners, a process that has been described elsewhere [3].

Traditional investigative tools, such as laboratory-based surveillance, do face challenges including but not limited to underdiagnosis and underreporting of illnesses, varying illness reporting structures, the increased use of culture-independent diagnostic testing, and resource availability [16,17]. While some of these challenges are mitigated by public health consumer complaint systems described earlier, the increased accessibility and use of novel data streams (NDSs) provide new information that may further aid in the detection and investigation of foodborne illness clusters. NDSs include nontraditional sources of information such as social media, web-based customer reviews, multimedia news (comment sections from news stories about clusters of illnesses), and private participatory reporting platforms (PPRPs) that actively collect data on food poisoning.

The availability of crowdsourced web-based information provides an opportunity for public health entities to potentially augment existing traditional foodborne illness surveillance systems or identify clusters not detected by traditional tools. The evolution of cell phones to more readily allow users to access the internet has made these NDSs even more accessible as a tool for the submission of consumer complaints. These NDSs, in the context of foodborne illness, consist of reports of consumer information related to food products that are relayed digitally through social media, search histories, customer reviews (ie, product and restaurant reviews), and PPRPs [18]. Some PPRP sites have the express goal of collecting data on people who believe that they have gotten sick from food. Information from these sources may be shared directly with public health agencies or may be mined by agencies in response to a signal.

NDSs can be integrated with traditional public health surveillance programs to catalyze or supplement investigation, and several examples of this exist at the local and state health department level. For instance, the Chicago Department of Public Health has used social media posts generated within jurisdictional limits to address consumer complaints of food poisoning and to target health inspections [19]. The New York City Department of Health and Mental Hygiene has similarly evaluated the use of customer-submitted restaurant reviews to detect outbreaks and target health inspections [20]. These are just a few documented examples of the use of NDSs by state and LHDs. No systematic use of these data sources has been documented at the federal level at this time.

**Figure 1.**
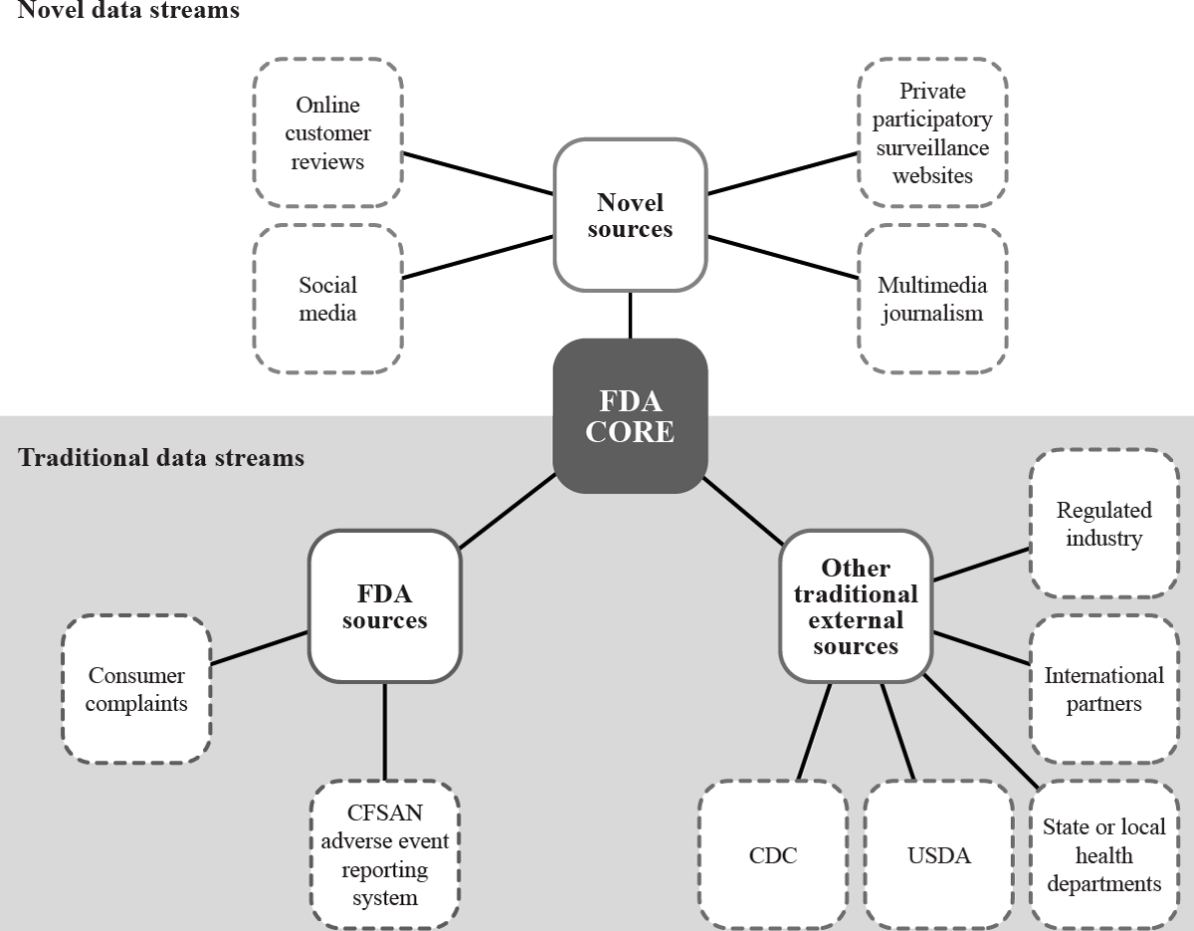
Examples of adverse event and illness cluster data sources evaluated by the US Food and Drug Administration Coordinated Outbreak Response & Evaluation (FDA CORE) Network. Multimedia journalism may also be considered a traditional data source in some cases. CFSAN: Center for Food Safety and Applied Nutrition; CDC: Centers for Disease Control and Prevention; USDA: US Department of Agriculture.

Reports of foodborne illness clusters can come from a variety of traditional and novel data sources (Figure 1). In this review, we (1) highlight key details of 3 recent adverse event clusters that were investigated by the FDA and were catalyzed or supplemented by NDSs, (2) discuss challenges and limitations associated with the current use of NDSs by the FDA, and (3) propose opportunities for improvement and the future use of NDS applications during foodborne illness surveillance and investigational activities conducted by the FDA.

## Three Case Studies

### Overview

To illustrate the use of NDSs during FDA investigations of foodborne illness clusters, the following 3 case studies describe recent incidents linked to dry breakfast cereal, ketogenic shakes, and plant-based crumbles, respectively. Each of these incidents provides a unique look at how these data streams were used by the FDA to identify or supplement investigations. There may be duplicate reports included in the analyses of these case studies, as reports may have been submitted by the same complaint through multiple points in FDA and to a given PPRP or other form of NDS. The following case studies are meant to highlight examples of this use and are not meant to be comprehensive reviews of the entire incidents.

### Dry Breakfast Cereal

On April 1, 2022, the New York Post published an article that highlighted an increase in illness reports mentioning a dry breakfast cereal product on a PPRP [21]. The article stated that at least 139 consumers reported vomiting and diarrhea after consuming the dry breakfast cereal of interest, which prompted an FDA investigation. After FDA CORE initiated an investigation, the PPRP shared complaint data with the FDA, which was reviewed by FDA CORE and used for an initial assessment of the incident. FDA’s medical evaluation of 470 PPRP reports received from January 1, 2022, to April 5, 2022, identified symptom onset data for 349 (74%) reports, most of which (96%) suggested nonrapid symptom onset (exceeding 30 min since consumption). In some cases, due to the nature of the reporting tool, these data sometimes omitted necessary epidemiologic information such as the date the person first reported feeling ill, food exposure date, and illness duration.

Thus, the investigation instead focused on reports directly submitted to the FDA. FDA consumer complaint reports collected between June 1, 2021, and May 10, 2022, that mentioned exposure to the dry breakfast cereal product of interest and explicit gastrointestinal (GI) symptoms were reviewed. Only one of the 120 consumer complaint reports submitted between June 1, 2021, and May 10, 2022, was received in 2021. The remaining 119 were submitted after April 1, 2022, the day the NY Post article was published. Of these 120 consumer complaints, many reported GI symptoms.

**Figure 2.**
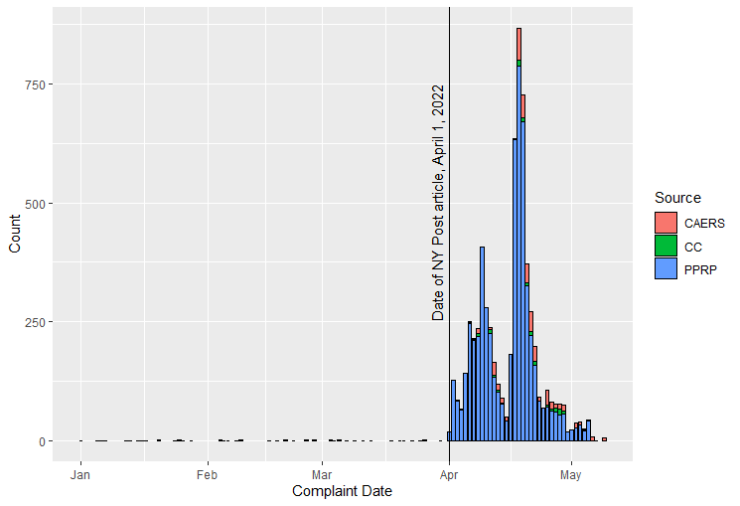
Distribution of adverse event reports received by various reporting platforms concerning dry breakfast cereal (2022). CAERS: Center for Food Safety and Applied Nutrition Adverse Event Reporting System; CC: Food and Drug Administration consumer complaints; NY: New York; PPRP: private participatory reporting platform.

Between June 1, 2021, and May 10, 2022, there were a total of 438 Center for Food Safety and Applied Nutrition Adverse Event Reporting System (CAERS) reports associated with the dry breakfast cereal product, 437 of which were submitted after April 1, 2022 (Figure 2). The FDA conducted an analysis of 406 CAERS reports that contained suspected cases with exposure to the breakfast cereal, explicit GI symptoms, and were received prior to April 30, 2022. Of 406 CAERS reports, only 160 had information on illness onset, most of which suggested symptom onset occurred 1‐24 hours after consumption. Overall, 558 FDA adverse event reports (120 consumer complaint and 438 CAERS reports) and 6272 PPRP reports of GI illness were associated with this incident.

In response to illness reports related to the dry breakfast cereal product, the FDA conducted preventive control inspections at 2 manufacturing facilities that produced the dry breakfast cereal of interest and at 1 product supplier in order to document manufacturing operations and identify potential deficiencies. An FDA investigation was also conducted at the corporate offices of the dry breakfast cereal brand in order to collect records pertaining to manufacturing processes, facilities, products, suppliers, quality control practices, and consumer complaint data. Concurrently, the Michigan Department of Agriculture and Rural Development initiated a preventive control inspection at another supplier to the brand. No significant observations were found during these inspections. FDA chemical and microbial analyses were completed on product and environmental samples. FDA and state partners also analyzed clinical and consumer product samples. However, no causative agent for this incident was identified. Evidence from this investigation indicated that this was, in fact, not a microbial outbreak related to dry cereal.

### Ketogenic Shakes

In March 2022, a PPRP shared with the FDA an analysis of complaints they received about Brand A protein or meal replacement shakes, a generic brand produced for Retailer A. The analysis demonstrated that there was an increase in complaints associated with Brand A when compared to other protein or meal replacement shake brands starting in January 2021. The PPRP provided all complaints to the FDA for Brand A protein or meal replacement shakes submitted between January 2021 and March 2022. A review of this information found that 28 of 32 reports specifically identified the ketogenic shake from Brand A, and the FDA initiated an investigation into this product. During the investigation, 6 additional complaints were received, culminating in a total of 34 complaints specifically identifying Brand A ketogenic shakes reported to the PPRP between January 2, 2021, and March 30, 2022. These PPRP reports were challenging to use as they lacked complete epidemiologic information, and specific product details, such as lot numbers, were not consistently available. Further, some of the data that was provided was unstructured and required manual review and extraction.

Additionally, reviews for the ketogenic shake product were extracted on March 24, 2022, from Retailer A’s website. This data also required manual review and data extraction. Between January 9, 2021, and February 2, 2022, 54 reviews contained text with complaints indicating an adverse event. Finally, FDA systems were queried for complaints related to the Brand A ketogenic shake over the previous 2 years. Between June 6, 2021, and April 8, 2022, 2 consumer complaints and 4 CAERS reports were received by the FDA. Complaints from all 3 data sources (FDA, consumer reviews, and PPRP complaints) were compiled, and a total of 93 were reviewed. Symptoms were varied but included mostly GI distress, and many reported a rapid onset of symptoms (<30 min). The number of reports received for Brand A was compared to the number for Brand B ketogenic shake to ascertain whether the amount of complaints was unusual. The Brand B ketogenic shake had nearly equivalent product labeling to the Brand A ketogenic shake, but the 2 brands did not share a manufacturer. Both brands were nationally distributed in the United States. The FDA CAERS database and the PPRP were also queried for complaints that specified Brand B during the same time frame. These Brand B queries returned no complaints from the PPRP and 1 complaint from FDA CAERS.

Closed retail samples of 2 flavors of Brand A ketogenic shakes and open and closed samples from a complainant’s home were collected. Chemical and sensory analyses were completed on all product samples collected by the FDA, and no unusual findings were identified. No public communications or regulatory actions resulted from this investigation. However, details of the investigation were shared with Retailer A, who had already discontinued distribution of the product based on poor web-based reviews and who further, in response to FDA notification, made the remaining product in their stores unavailable for purchase. Ultimately, laboratory testing did not detect any specific undeclared ingredients or toxins as a causative agent for these adverse events, but the investigation did highlight potential consumer intolerances to some declared ingredients used in ketogenic food products.

### Plant-Based Crumble Product

In June 2022, the FDA received consumer complaints and adverse event reports referencing GI illness suggesting abnormal liver function (eg, jaundice and dark urine) that may have been attributable to the consumption of a plant-based crumble product produced by Brand C. As of June 23, 2022, 20 CAERS reports and 13 consumer complaints had been evaluated. Although some complainants reported several Brand C products, all complaints specified the plant-based crumble product. Most reported GI and liver-associated symptoms. This incident became heavily publicized as reports pertaining to the adverse events were reported digitally. Additionally, consumers reported adverse health outcomes to various other sources, such as state reporting mechanisms, a PPRP, and directly to Brand C. As of October 18, 2022, this incident included 393 (177 consumer complaints and 216 CAERS reports) adverse event reports in 39 states with 133 hospitalizations and zero deaths. Estimated illness onset dates ranged from April 19, 2022, to September 4, 2022.

Closed and open samples of Brand C plant-based crumbles product were sampled from complainants’ homes. Closed and undistributed product samples were also obtained from Brand C’s warehouses. Both chemical and microbiological analyses were performed, but there were no significant findings. However, in 2024, the FDA made a public posting noting that tara flour, an ingredient used in this product, did not meet the Generally Recognized as Safe standard, so it was an unapproved food additive [22]. The FDA issued a web post on the investigation and posted the company recall on the FDA recalls page [23,24]. The FDA further performed several domestic inspections related to the incident and 1 foreign inspection, though none of these actions provided evidence of contamination.

During this investigation, the FDA also reached out to the PPRP to examine any reports they had received regarding this product. The website had received 24 complaints associated with Brand C products from April 4, 2022, to October 18, 2022, 7 of which mentioned the product of interest (plant-based crumbles). Of these 7 complaints, 4 reported GI symptoms and 3 reported various liver-associated ailments. Records from the PPRP were obtained to rule out other vehicles of interest, such as other Brand C products and products from a separate company, Brand D. Although 8 other products were mentioned, none were mentioned more than twice. Further, the PPRP had only received a single complaint for Brand D, which described nausea and vomiting. Based on these reports and the data from FDA systems, other Brand C and Brand D products were excluded as not likely vehicles of illness for this incident.

## Discussion

### Overview

The use of NDSs to identify adverse event signals or supplement FDA investigations of foodborne illness clusters may offer opportunities such as earlier detection of clusters and vehicle confirmation as seen in the case studies described earlier. However, data collection from NDSs also presents challenges that may reduce their utility during ongoing incident investigations. The advantages and challenges associated with the use of NDS data are discussed in the following section, along with a summary of future steps to better harmonize public health efforts.

### Advantages

Traditional, laboratory-based methods of foodborne disease surveillance are largely reliant on the detection of foodborne illnesses via information collected at the clinical level, such as laboratory tests and health care provider reports [25]. These signals are dependent on ill persons seeking medical care, yet some research indicates that fewer than 15% of individuals with self-suspected foodborne illness seek medical attention or report to an LHD [25]. The most recent Foodborne Disease Active Surveillance Network (FoodNet) population survey supports this, noting that only 16.9% of respondents with an acute GI illness sought medical care [26]. Further, most episodes of foodborne illness are self-limiting, and only a small percentage of illnesses in the United States are estimated to result in hospitalization [2]. Once a case of foodborne illness is detected, associated epidemiologic information is gathered by trained epidemiologists, which ensures collected information is detailed and accurate. However, national mandatory reporting of foodborne illnesses is limited to a select set of diseases [27]. While this list includes most well-established foodborne pathogens, it is not as useful for illnesses without a known etiology. Notification of the public health system to new illnesses is also slow, as lag times often range from 2 to 4 weeks or more, depending on the etiology, time of year, and laboratory testing capability. As discussed, some of these limitations are mitigated by various consumer complaint-based systems that are not limited by specific etiology and do not require a person to seek medical care. This is particularly important for localized outbreak detection.

NDSs have the potential to address gaps that exist in traditional foodborne illness surveillance methods and empower consumers. For example, data from these systems can alert public health agencies to foodborne illnesses in situations where medical care is not obtained (eg, due to financial reasons, lack of perceived necessity). NDSs may also provide early signals for foodborne illness clusters that would otherwise be slowed due to expected lag times in case reporting or unforeseen delays. Common barriers to traditional reporting, such as cost, time, access, or awareness, may also be avoided by consumers who use NDSs. In the United States, mechanisms to report an adverse event directly to a public health entity vary across the state and federal levels, while NDS companies have varying user-friendly formats for accepting consumer complaints. Therefore, the generation of an adverse event report on a PPRP or a review on a product webpage may be more intuitive, simpler, and less time-consuming for the public in some situations.

The broad reach of NDSs strengthens their ability to catalyze public health investigations. The larger number of associated complaints can also contextualize reported symptoms, which is useful in situations where the pathogen or hazard is emerging and case symptomology is unknown. One example of this is the ketogenic shake investigation, in which complainants described a rapid onset of short-lived symptoms after consuming the product, making it more unlikely for complainants to seek care under these circumstances. Despite the low volume of related complaints reported to the FDA, which typically would not rise to the level of an investigation, an FDA investigation was triggered due to the number of complaints reporting a similar product or symptomology within product reviews and PPRP reports.

A signal received via NDS of a suspected illness related to an FDA-regulated product may allow the FDA to evaluate consumer risk using investigative tools such as traceback if the incoming data includes necessary details about the product. Traceback helps ascertain if illnesses are related to a common source, which leads to further investigation and, potentially, product actions. Details within NDS reports may help to target firm inspections, product sample collection, and product testing, all of which can inform compliance actions. Retailers may also use information from NDSs as an early alert to a poor-quality product or one that may be causing adverse reactions among consumers, such as in the example provided with ketogenic shakes. This unsolicited information allows retailers to internally initiate actions to protect consumers prior to a manufacturer-initiated product recall or the attention of regulatory and public health agencies. Traceback information obtained from NDSs may also help to rule out products not linked to adverse events. Although NDS data can inform industry and regulatory investigations, the source for NDS reports may be anonymous; therefore, the veracity of the data should be considered during decision-making.

### Challenges

An overarching limitation of NDSs stems from the quality of available data. Consumer complaints made to the FDA use forms that incorporate standardized epidemiologic data elements, including those identified by the Council to Improve Foodborne Outbreak Response [27]. These forms also collect details used to assess complaint trends and detect potential food safety issues. NDSs do not reliably capture key data elements essential for public health investigations such as those outlined in Table 1, which includes an expanded list of data elements in addition to those put forth by the Council to Improve Foodborne Outbreak Response. As NDSs often obtain information for other purposes, data gaps are expected and can hinder public health investigations. For example, epidemiologic information is used by investigators to identify a common etiologic agent, which is critical in the absence of clinical validation. Without epidemiologic information, the cause of a food safety issue is more challenging to identify, and the scope of product testing is significantly more resource intensive. Product and exposure information are also necessary to identify a common source of illness and inform traceback investigations. A lack of actionable data inhibits public health response, which was illustrated during the 3 investigations described here.

Another limitation associated with NDS systems is that the data are often unstructured. Information from complainants is typically in the form of free-text entry (eg, social media posts, product reviews, web-based comments), which currently require manual review to extract key data elements. This process is resource-intensive, subject to human error, and has limited feasibility for large numbers of posts or comments. Automated data review may reduce the burden of manual review in the future, but specialized tools are expensive investments for ad hoc analyses, and varying data sources may necessitate different technical approaches. Similar to traditional outbreak data sources, NDS reports may also be duplicated across reporting platforms, as individuals may submit complaints on multiple NDS platforms, multiple complaints on one platform, and directly to the FDA (Figure 1). Some NDS platforms do minimize report duplication with requirements for users to sign into an account, to have verified purchases to leave a review, or by tracking the IP address of the user. However, evaluation of complaint uniqueness for enumeration across platforms may require manual deduplication or simply not be possible.

**Table 1.** List of key data elements for actionable public health response for collection by novel data systems, expanded and modified from the Council to Improve Foodborne Outbreak Response [27].

Data type	Data element
Epidemiologic	Illness onset date and time Symptoms Duration of symptoms Care-seeking behavior Suspected exposure of interest Exposure date Other recent exposures (past 7 days)
Product	Photo of product packaging Brand Best buy date or best if used by date Lot code Product description on label Purchase location and purchase date Manufacturer, distributor information
Complainant’s contact information	Name Phone number Email address
Affected individual’s contact information (if not the same as the complainant’s)	Name Phone number Email address
Affected individual’s demographic information	Age Gender State of affected individual’s residence
Method of report	Date of complaint Type of platform Method of complaint

Ultimately the structure, breadth, and utility of information available in an NDS are dependent on the purpose of the platform. Certain NDSs, such as web-based reviews and social media posts, share information publicly, though not explicitly for public health goals. Due to this, food safety complaints made on these platforms usually lack critical data elements but have the potential to provide contextual information that can catalyze or supplement an investigation. PPRPs do have a public health goal, and compared to official government complaint systems, they may be easier to find and use, which makes them attractive options to complainants. However, although these sites may be easier to use, the data captured may be incomplete or of lower quality than data collected from traditional complaint systems. Public health and regulatory officials must keep these limitations in mind when considering NDSs in an investigational context.

When a signal for an adverse event is received, public health investigators review the reported symptoms, in conjunction with laboratory diagnoses, to determine an etiology and define cluster criteria (ie, develop a case definition) [27]. With NDSs, limited epidemiologic information may result in nonspecific symptomology. Alternatively, investigators may rely on medical records and laboratory documentation to accumulate evidence in search of a possible etiology, but complainant contact information is not often included in NDS reports, which prevents investigators from obtaining follow-up information. Thus, reports from NDSs must be grouped based on available epidemiologic features, which results in a broad case definition, extensive record requests, and resource-intensive review by investigators. Therefore, biological plausibility and medical review should be considered when refining a case definition and evaluating likely exposures when using NDSs.

Evidence has shown that media attention, particularly TV news coverage, can lead to an increase in adverse event reporting during a health scare [28,29]. This would also affect reporting to NDSs, which could see greater impacts given their ease of reporting, particularly if an NDS uses tools such as social media to share information on the incident. This effect was illustrated during the dry cereal incident, as complaints to both NDSs and FDA increased dramatically after newspaper reporting (Figure 2). One possible further complication of media attention is the amplification of unreliable and spurious signals that leads to stress on public health response entities, who already have limited resources.

Many actors are involved in multistate investigations, each with unique roles and challenges. One of these challenges is identifying proper medical and epidemiologic experts to evaluate data collected from PPRP or other NDS sources. CDC, for example, has investigational programs aligned to specific pathogens, so it can be difficult to identify which group would provide expertise during an investigation involving an unknown etiology.

As NDS platforms are not managed by established public health systems, information sharing with entities that have authority to take public health action is limited. Although some public health agencies may partner with third-party NDS platforms, subscribe to their services, or survey their data, NDS data may not be automatically escalated to a public health authority, which may not always be clear to the public when submitting their report. Also, some NDS companies may require public health agencies to pay to access complaint data. In addition, NDS platforms vary in the user information they collect, which can limit the ability to conduct follow-up with ill persons. It is also important to note that these NDS systems may divert ill people away from submitting reports to established public health reporting and surveillance systems. People who report their illnesses on these NDS platforms may assume that their reports will automatically be shared with authorities, which is not the case. In addition, submitting complaints to NDS systems may give people the false expectation that the information they provide will be actionable by public health agencies.

One reason that laboratory results can be confirmed relatively quickly in traditional outbreaks is that the etiological agent is known, and multiple laboratories are often able to perform and analyze testing results. Conversely, NDS-detected outbreaks often have an unknown etiological agent, which requires broad, instead of targeted, testing and often takes longer to return results.

Similar to limitations discussed regarding epidemiologic data, information collected from consumers through NDSs may lack critical elements needed for product traceback, such as product and purchase information. Although photos can be shared of a product, photos and product descriptions obtained from consumers do not consistently provide information on lot numbers, purchase dates, or purchase locations. Using a phone to submit complaints, while convenient, may limit the amount of information that people are able to submit. This information can aid regulatory authorities to conduct traceback, as some products may have the same name or label but have been produced or grown at different locations. Purchase information may also inform traceback investigations through identification of the manufacturer; however, these details are not consistently captured from NDSs or from traditional outbreak data sources. Ultimately, the lack of consistent product and purchase information may hinder the identification of a specific implicated product for further industry or regulatory action.

### Opportunities

While NDSs have been helpful at the local level in identifying establishment-based illness clusters or targeting limited resources for inspections, foodborne investigations at the federal level focus on multistate outbreaks or vehicles that enter interstate commerce [7,20,28]. In these situations, etiologic agents are generally not related to ill workers or conditions at a retail establishment, but rather issues further up the supply chain. Similar to traditional outbreak data sources, NDSs reports may be of assistance during FDA investigations to identify signals related to branded or packaged products that are easily identified by the consumer, as opposed to loose items, such as produce, with no clear or easily identifiable brand or manufacturer. NDSs are also most likely to identify incidents that involve a rapid onset of symptoms, as with the ketogenic shakes investigation, or unusual symptoms, as in the plant-based crumble product investigation.

Signal detection and data collection through NDSs will likely become more prevalent. Therefore, it is incumbent upon public health agencies to determine the best ways to use this data, integrate it into established signal detection systems, and potentially collaborate with platform hosts. There is also a need to establish a process, once a signal is detected via NDS, where ill individuals can be interviewed with a standardized questionnaire that can be used to further evaluate potential exposures and etiologies. NDS providers should consider adding the noted critical data elements (Table 1) to existing data captured by their sites or provide public health officials with contact information to follow up with complainants. CDC and state health departments or LHDs can play an important role in coordinating patient interviews and epidemiological data collection, including medical records. In the future, the further development of tools for natural language processing, website scraping, and optical character recognition technology (software to extract text from an image) may also be useful when applied to NDSs for signal detection or to supplement ongoing outbreak investigations.

## Conclusions

NDSs may enable quicker signal detection in certain situations compared to traditional surveillance methods for foodborne illness clusters attributable to unknown agents. Recently, NDSs have provided supplementary data during traditional investigations and sparked investigations into previously undetected signals, which may or may not have provided enough information for an actionable public health response. While PPRPs and product reviews are the focus in this paper, future signals may come from other NDSs, such as web-based social media platforms. To effectively integrate these data into public health surveillance and response, working protocols could be developed to help agencies better collect, surveil, and assess this data. NDSs with a focus on public health should also do their best to collect critical epidemiologic, product, and clinical data, enhancing their utility as a resource for public health agencies.

## Abbreviations

CAERS: Center for Food Safety and Applied Nutrition Adverse Event Reporting System
CFSAN: Center for Food Safety and Applied Nutrition
CORE: Coordinated Outbreak Response & Evaluation
FDA: Food and Drug Administration
GI: gastrointestinal
LHD: local health department
NDS: novel data stream
PPRP: private participatory reporting platform
